# Effects of date palm pollen on fertility: research proposal for a systematic review

**DOI:** 10.1186/s13104-017-2697-3

**Published:** 2017-08-01

**Authors:** Fatemeh Abdi, Nasibeh Roozbeh, Amir Mohammad Mortazavian

**Affiliations:** 1 Student Research Committee, Nursing and Midwifery Faculty, Shahid Beheshti University of Medical Sciences, Tehran, Iran; 2 Mother and Child Welfare Research Center, Hormozgan Universiy of Medical Sciences, BandarAbbas, Iran; 3 Department of Food Technology, Faculty of Nutrition Sciences and Food Technology/National Nutrition and Food Technology Research Institute, Shahid Beheshti University of Medical Sciences, Tehran, Iran

**Keywords:** Date palm pollen, Sperm, Fertility, Ovary

## Abstract

**Objective:**

Over 10–15% of couples in different countries are infertile. Male infertility is a contributing factor and the only cause of infertility in respectively 50% and 20–30% of all cases of infertility. According to previous research, micro-elements isolated from date palm pollen (DPP), e.g. estrogen and sterols, may enhance male and female fertility. DPP has also been reported to improve sperm parameters including sperm motility and viability, acrosome reaction, and lipid peroxidation. This article may justify the need for a future systematic review and meta-analysis about the effects of DPP on the reproductive system and DPP’s ability to enhance fertility. It will then describe the methodology of such a study.

**Main text:**

A comprehensive search of relevant randomized and quasi-randomized controlled trials will be performed in MEDLINE, EMBASE, Web of Science, Cochrane Central, ProQuest, and Google Scholar databases. Two authors will independently assess the eligibility of the studies and consult the third author in cases of disagreement. The risk of bias of the randomized controlled trials and animal studies will be evaluated using the Cochrane risk of bias tool and the Systematic Review Centre for Laboratory animal Experimentation (SYRCLE) risk of bias tool, respectively. This study will raise no ethical issues as it will review the findings of previous research. The results are intended to be published in a peer-reviewed medical journal.

## Introduction

Since any impairment in the reproductive system is generally considered as a disease, infertility is a negative concept in most cultures. According to the World Health Organization (WHO), infertility is a couple’s inability to become pregnant after at least 1 year of regular, unprotected sexual intercourse [1]. About 10% of infertilities have no particular reason [2] and 12–18% of women fail to get pregnant after 12 months, or cycles, of trying [3]. Male infertility rates around the world range between 2.5 and 12% and over 30 million men worldwide are infertile. Male infertility is a contributing factor and the only cause of infertility in respectively 50% and 20–30% of all cases of infertility [4, 5]. Low sperm count and/or quality is present in up to 90% of couples with fertility problems [6].

Complementary therapies for infertility have received growing attention during the recent years and various antioxidants, nutritional approaches, and medicinal plants have been proposed for the treatment of fertility problems in infertile and sub fertile couples [7–9]. A prospective cohort study in the US estimated the prevalence of using alternative and herbal medicines by couples with fertility problems at 29 and 17%, respectively [10]. Due to increasing interest in the health benefits of medicinal plants, a wide range of herbal products has been produced and distributed in the global health market [11]. The benefits of medicinal plants in the treatment of sperm abnormalities have been attributed to their antioxidant, anti-inflammatory, and venotonic activity along with their contents that promote sperm production and increase blood testosterone levels [12].

Date palm pollen (DPP), the male reproductive dust of palm flowers, has long been used as a dietary supplement to increase libido and improve fertility in both women and men. DPP is known to contain a variety of compounds including amino acids, fatty acids, flavonoids, saponins, and sterols [13]. Owing to its contents, e.g. estrogen-like compounds, sterols, estrone-like compounds, and steroidal saponin glycoside, DPP is believed to increase female fertility [14]. Moreover, since estrogen has been recently found to contribute to the regulation of spermatogonial stem cells and male reproductive tissues with estrogen receptors, DPP, which contains estrogenic gonad-stimulating compounds, estrogen, sterols, and other beneficial micro-elements, can help treat male infertility [15]. According to previous research, DPP can enhance sperm motility and viability, acrosome reaction, and lipid peroxidation [16]. Several experimental studies, along with a few clinical trials, have evaluated the effects of DPP on the reproductive system. Compared to previous systematic review, this study may demonstrate the impact of DPP on male and female reproductive system and may justify the need for a future systematic review and meta-analysis about the effects of DPP on the reproductive system and DPP’s ability to enhance fertility. It will then describe the methodology of such a study.

## Main text

### Research questions

1. Is DPP effective to the treatment of both male and female infertility?
2. Does DPP improving reproductive parameters?
3. Does DPP preventing the testicular and ovarian dysfunction?

### Materials and methods

#### Registration and methodology

The study protocol was registered in the International Prospective Register of Systematic Reviews (PROSPERO) at the National Institute for Health Research. Registration Number in PROSPERO is CRD42016054297. The guidelines of PRISMA-P (Preferred Reporting Items for Systematic Review and Meta-Analysis Protocols) were followed while reporting the study protocol.

#### Timeline

After defining search terms, a pilot search was performed and a data extraction form was developed. A full search was scheduled to start in the third week of November, 2016 and extended to the latest depending on the date of publication of this protocol.

### Eligibility criteria

#### Types of participants

Human participants

- Couples who failed to conceive after over 12 months of unprotected sexual intercourse;
- Infertile 20–65-year-old men who used DPP (irrespective of treatment duration) to enhance sperm parameters and testicular function. Testosterone, estradiol, follicle stimulating hormone (FSH), and luteinizing hormone (LH) measurements and spermatograms were used to assess the reproductive system.
- Infertile 15–50-year-old women who used DPP (irrespective of treatment duration) to improve the reproductive function. Estrogen, progesterone, FSH, and LH levels were measured to assess the reproductive function.

Animal participants

- Fertile and infertile adult rats, mice, rabbits, and hamsters which received DPP for improving fertility (regardless of sex or race).
- Serum FSH, LH, testosterone, and estradiol levels were measured to assess the reproductive system of male animals.
- Serum estrogen, progesterone, FSH, and LH levels were measured to assess the reproductive system of female animals.

#### Types of intervention(s)/phenomena of interest

This review will include studies that administered DPP (through any routes) for the treatment of reproductive problems. Treatment with DPP will be compared with no treatment group, treatment with placebo, and treatment with other agents. Studies will be selected if they used a standard diet during the treatment period.

#### Type of outcome measures

Primary outcomes

1. Response rate to treatment measured through spermatograms and hormonal evaluations;
2. Changes in total sperm count, concentration, and motility measured through spermatograms;
3. Testicular function evaluated by measuring testosterone, estradiol, FSH, and LH levels;
4. Ovarian function evaluated by measuring estrogen, progesterone, FSH, and LH levels;
5. Effects of DPP on ovarian follicles determined through ultrasound; and sperm quality, testis, prostate, epididymis, and seminal vesicle evaluations as well as follicles and ovarian assessments in animal studies.
6. Pregnancy or live birth rates after treatment by DPP.

Secondary outcomes

1. Effects of pretreatment with DPP in decreasing sperm abnormality evaluated through spermatograms;
2. Incidence rate of adverse events caused by treatment (evaluated based on the Common Terminology Criteria for Adverse Events); and
3. Effects of DPP on ovary and testis weight in animal studies (evaluated through biopsy).

#### Types of studies

Randomized and quasi-randomized controlled trials published between 2000 and 2016 will be included. Observational and qualitative studies, along with letters to the editor, case series, and case reports will be excluded. No language limitations will be imposed during the search.

#### Search strategy

A comprehensive literature search, with no language restriction, will be performed in reliable electronic databases including MEDLINE, EMBASE (Scopus), Cochrane Central Register of Controlled Trials (CENTRAL), ProQuest, Google Scholar, and CINAHL (via EBSCO).

The reference lists of studies extracted from the primary electronic search will be used to identify additional relevant studies. The citations in the extracted studies will also be evaluated using the Web of Science. The following key terms and their combinations (made by applying Boolean operators AND and OR will be used during the search: DPP, pal pollen, date palm pollen, Phoenix dactylifera pollen, male infertility, sperm, testicular function, female infertility, and ovarian. Key journals, government reports or scientific research reports, and article abstracts presented in conferences will also be searched manually.

### Data collection and analysis

#### Selection of studies

Two authors (FA, NR) will independently evaluate the titles and abstracts of the initially included studies. They will also extract the full texts of studies meeting the inclusion criteria and check to prevent the inclusion of duplicate publications of a single study. The authors will extract data from all eligible studies and record the extracted data in the relevant forms.

#### Quality appraisal

Clinical trials will be included and standards reporting of these studies will evaluate by CONSORT (Consolidated Standards of Reporting Trials) checklist. The CONSORT checklist consists of 24 items scored as 0 or 1 [17]. Moreover, animal studies will be included if they were reported based on the Animal Research: Reporting of in vivo Experiments (ARRIVE) guidelines. The ARRIVE checklist uses 20 items to summarize the minimum information required during the reporting of animal studies. Each item is scored as 0 or 1 [18]. In order to ensure the quality of the included papers, studies that score below 60% on either checklist will be excluded.

Two authors will independently evaluate the risk of bias in randomized controlled trials and animal studies using the Cochrane Risk of Bias tool [17] and the Systematic Review Centre for Laboratory animal Experimentation (SYRCLE) risk of bias tool, respectively [19]. These authors will score the risk of bias in each domain and the overall risk will be reported as a percentage. The third author will be consulted to resolve any cases of disagreement. The results of these assessments will present in a risk of bias Table.

#### Data extraction

The authors will independently extract data, including type of study, number of participants (either humans or animals), type and dose of administered interventions, outcomes, and risk of bias, from the eligible studies and record the collected data in a researcher-made form. They will then check the accuracy of the collected data and enter the data into Review Manager. Cases of disagreement will be resolved through discussion and consultation with the third author if necessary [20].

#### Data synthesis

Whenever possible, quantitative data will be pooled in statistical meta-analysis using STATA software. Double data entry will be performed for all results. Effect sizes will be presented as odds ratios (for categorical data) and weighted mean differences (for continuous data). Their 95% confidence intervals (CIs) will then be calculated for analysis. In order to evaluate clinical heterogeneity, the populations, interventions, and outcomes of different studies will be considered. Statistical heterogeneity will be investigated by calculating the I2 statistic, and if this indicates a high level of heterogeneity among the studies included in a meta- analysis, a random effects meta-analysis will be performed for the total summary. Separate meta-analyses will be conducted on animal and human studies and the obtained results will be compared. Subgroup analyses, e.g. among different study types, studies with different control groups, studies on different types of infertility, and studies evaluating different outcomes, will also be performed when adequate numbers of studies are available.

## Discussion

This protocol will be the latest review of the available literature, including large randomized controlled trials and animal studies, about the effects of DPP on the reproductive system. This is the first systematic review that will perform based on human and animal studies, separate meta-analyses will be performed on studies and the results will be compared. Due to the increase of infertility in recent years, adverse effect of chemical treatment and the attention to effective herbal drugs for infertility, herbal medicine can be an appropriate alternative to conventional medications [8]. If the efficacy of DDP will demonstrate in the present study, given to the cost of infertility treatment, this medicine could be useful for health care system especially in low income countries.

## Limitations

- In this research it is possible that the primary and secondary infertility will be effect on the response rate.
- Maybe some studies do not meeting the eligibility criteria.

## Abbreviations

PROSPERO: International Prospective Register of Systematic Reviews
DPP: Date Palm Pollen
SYRCLE: Systematic Review Centre for Laboratory animal Experimentation
PRISMA-P: Preferred Reporting Items for Systematic Review and Meta-Analysis Protocols
WHO: World Health Organization
FSH: Follicle Stimulating Hormone
LH: Luteinizing Hormone
CONSORT: Consolidated Standards of Reporting Trials
ARRIVE: Reporting of in vivo Experiments
